# A Retrospective Study of Rare Listeria Meningoencephalitis in Immunocompetent Children in China

**DOI:** 10.3389/fneur.2022.827145

**Published:** 2022-03-02

**Authors:** Tingting Mo, Fang Wu, Xiangjun Dou, Dong Wang, Han Xia, Xia Li

**Affiliations:** ^1^Department of Pediatric Neurology, Xi'an Children's Hospital, Xi'an, China; ^2^Department of Scientific Affaires, Hugobiotech Co., Ltd., Beijing, China

**Keywords:** *Listeria (L.) monocytogenes*, meningoencephalitis, children, immunocompetent, next-sequencing

## Abstract

**Objectives:**

Listeria meningoencephalitis (LMM) is very rare in healthy children. We aimed to assess the clinical features, differential diagnosis, treatment options, and outcomes of LMM in immunocompetent children through a retrospective study.

**Methods:**

The clinical symptoms, laboratory findings, imaging features, antibiotic use, and metagenomic next-generation sequencing (mNGS) results of the cerebrospinal fluid (CSF) were obtained from immunocompetent children who were diagnosed with LMM and admitted to the Xi'an Children's Hospital from May 2018 to July 2020.

**Results:**

The data from 8 immunocompetent children were retrospectively analyzed in this study. The cohort included data from 5 males and 3 females who were aged from 1 year and 7 months to 16 years and 6 months. A total of 4 patients had chilled food before onset. The complications included hyponatremia (3/8), hydrocephalus (2/8), and hemophagocytic syndrome (1/8). In total, 8 patients were diagnosed with *Listeria monocytogenes* by positive CSF culture or mNGS results. The positive rate of CSF culture was 62.5% (5/8). A total of 5 patients conducted CSF mNGS, and the results of the mNGS were positive in 4 patients (80%, 4/5) and suspected in 1 patient. A total of 7 patients changed their therapeutic regimen to combined antibacterial therapies that included linezolid and meropenem (5/8), or ampicillin and meropenem (2/8). A total of 5 patients had favorable outcomes (Glasgow Outcome Scale, GOSE = 5) while two patients had unfavorable outcomes (GOSE = 1) and were complicated with hyponatremia and hydrocephalus.

**Conclusions:**

Listeria meningoencephalitis (LMM) can occur in children with normal immune function and is commonly mistaken for other central nervous system infections. *L. monocytogenes* can be quickly and accurately detected by mNGS. Hyponatremia and hydrocephalus may indicate unfavorable outcomes.

## Introduction

Listeriosis is the third leading cause of death among major pathogens that are commonly transmitted by *Listeria monocytogenes-*contaminated food such as raw meats, milk, and ready-to-eat foods (1, 2). *L. monocytogenes* typically causes invasive infections (e.g., sepsis, meningitis, and congenital listeriosis) in newborn children, pregnant women, the elderly, organ transplant recipients, and patients with primary or secondary immunodeficiency (1–3). The incidence of listeria infection has risen sharply in developed countries since the 1980s (4). During the 21st century, the rates of listeriosis have ranged from 0.10 per 100,000 people to 0.98 per 100,000 people in developed countries (3, 5). The main entry site for listeria infection is the gastrointestinal tract through contaminated water or food (6). Bacteria invade the gut epithelial cells and cross the intestinal barrier to enter the bloodstream. The bacteria can infiltrate the brain from the blood or through the peripheral nerves, yet the precise mechanisms of infection remain to be fully understood (7).

Listeriosis rarely occurred in immunocompetent children beyond the neonatal stage. The clinical symptoms of *L. monocytogenes* meningoencephalitis (LMM) are similar to those from other causes, and so the misdiagnoses occur frequently in non-susceptible patients who are not immunodeficient in the early stage of the disease. In this study, we report on 8 cases of LMM in healthy children in which we evaluated the clinical features, differential diagnosis, treatment options, and outcomes.

## Materials and Methods

### Ethics Statement

This study was approved by the Ethics Committee of the Xi'an Children's Hospital. The patients were recruited under written informed consent obtained from the parents or legal guardians.

### Patient Recruitment

The data from children diagnosed with *L. monocytogenes* from May 2018 to July 2020 at Xi'an Children's Hospital were retrospectively analyzed. The diagnosis of LMM was based on the following criteria: A). clinical characteristics, including irritability, poor feeding, fever, seizures, fontanel bulge, neck stiffness, vomiting, and headache, B). elevated white blood cell and protein levels, low glucose concentrations in the cerebrospinal fluid (CSF), and a low CSF to blood glucose ratio (8), C). positive *L. monocytogenes* in the blood, CSF culture, or next-generation sequencing (NGS) of the CSF, D). primary immune deficiency based on immunological testing. Patients were excluded from the analysis if they were less than 1 year old and had a history of recurrent upper respiratory infections (>7 times per year) and lower respiratory infections (>3 times per year). Patients with organ transplants, cancer, and those receiving immunosuppressive drugs were also excluded from the analysis.

The clinical records were reviewed as well as the data extracted including patient demographics (e.g., sex and age), the clinical course of disease (e.g., clinical symptoms and signs, laboratory examination results, CT or MRI features, antibiotic therapy, complications, efficacy, and treatment outcomes.

### Specimen Processing and Metagenomic NGS (mNGS)

A total of 2–3 ml of CSF was collected by lumbar puncture, stored at −20°C, and delivered to Hugobiotech Co., Ltd. (Beijing, China) *via* air transport for analysis. Genomic DNA was extracted from the samples and sequenced on the Nextseq 550 platform (Illumina, San Diego, United States). High-quality sequencing data were generated by filtering out adapter, low quality, low complexity, and short reads. The human host DNA reads were also filtered by alignment to a human reference database (hg38). The remaining data were aligned to the Microbial Genome Databases (ftp://ftp.ncbi.nlm.nih.gov/genomes/) using Burrow-Wheeler Aligner software (9). The alignment results were used to calculate the coverage and depth of each species and presented the microbial composition of the samples.

## Results

### Clinical Characteristics and Examination

Our study focused on *L. monocytogenes* infections in 8 children that included 5 boys and 3 girls with no previous medical history. The median age of onset was 5.7 years. The clinical characteristics of all of the subjects are summarized in Table 1.

**Table 1:** The clinical characteristics of the immunocompetent children with LMM in this study.

**Patient**	**Gender**	**Age of onset**	**District**	**Inducement**	**Symptoms and signs**
1	M	1y, 7m	Village	Chilled food	Fever, vomit, irritable, neck stiffness
2	M	2y, 4m	Town	Uncertainty	Fever, torpid
3	M	13y, 9m	Village	Chilled food	Fever, vomit, headache, seizure
4	F	2y	Village	None	Fever, vomit, headache, torpid
5	M	5y, 3m	Village	Uncertainty	Fever, vomit, headache, torpid, neck stiffness
6	F	2y, 2m	Village	None	Fever, torpid, neck stiffness
7	M	2y, 7y	Village	Chilled food	Fever, vomit, seizure, hypologia, coma, neck stiffness
8	F	16y, 6m	Town	Chilled food	Fever, vomit, headache, drowsiness abdominal pain

*LMM, L. monocytogenes meningoencephalitis; M, male; F, female; y, years; m, months*.

Initial laboratory investigations showed that the white blood cell (WBC) counts were differentially elevated in 5 patients (the critical value of peripheral WBCs is 5–12 × 10^9^/L). The neutrophil counts and C-reactive protein (CRP) levels were increased in all cases and the levels of procalcitonin (PCT) were elevated in 7 patients. A total of 3 children were complicated with hyponatremia (115–124 mmol/L). Immunological screening of immune cells and complement levels were normal in all cases. Lumbar puncture was performed at 4 to 12 days from the date of onset. The limpid CSF revealed monocyte predominance, decreased glucose levels (glucose CSF/blood ratio <0.5), and elevated protein levels (Table 2).

**Table 2:** Laboratory examinations and brain imaging in the patients.

**Patient**	**Peripheral blood findings**					**CSF findings**				**Brian MRI/CT**
	**WBC count × 10^**9**^/L**	**Neutrophil cell count × 10^**9**^/L**	**CRP (mg/L)**	**PCT (ng/ml)**	**Na+ (mmol/L)**	**WBC count × 10^**6**^/L**	**Predominant cell**	**Protein concentration (g/L)**	**Glucose CSF/blood ratio**	
*1*	*23*	*17.6*	*37.6*	*3.15*	Normal	*2,046*	M	*883*	*0.29*	Abnormal signal in cerebral cortex and subcortical
*2*	6.91	5.13	*17.36*	*2.25*	Normal	*398*	M	*370.1*	*0.37*	Normal
*3*	*12.28*	*11.48*	*12.5*	0.05	Normal	*879*	M	*920.1*	*0.43*	Normal
*4*	*13.7*	*9.8*	*87.28*	*0.45*	Normal	*434*	M	*725*	*0.17*	Brain sulcus widening
*5*	*16.32*	*11.83*	*40.35*	*1.15*	*123*	*376*	P	*471.6*	*0.38*	Normal
*6*	*15.57*	*10.41*	*44.01*	*0.31*	Normal	*122*	M	*518*	*0.37*	Normal
*7*	7.2	6.2	*268.45*	*7.02*	*124*	*1,352*	M	*15,638.5*	*0.38*	Hydrocephalus, parenchymal edema
*8*	8.16	6.51	*64.74*	*0.2*	*115*	*3,083*	M	*4,737*	*0.1*	Hydrocephalus, parenchymal edema,

*WBC, white blood cells; CRP, C-reactive protein; PCT, procalcitonin; CSF, cerebrospinal fluid; M, Monocyte; P, polykaryocyte. Normal ranges of peripheral blood WBCs, neutrophil cells, CRP, PCT, plasma sodium, WBCs and protein concentration of CSF are 5-12 × 10^9^/L, 0.6-7.5 × 10^9^/L, 0–3 mg/L, 0–0.05 ng/ml, 130–150 mmol/L, 0–15 × 10^6^/L and 200–400 g/L, respectively. Abnormal values indicated with italic*.

**Figure 1:**
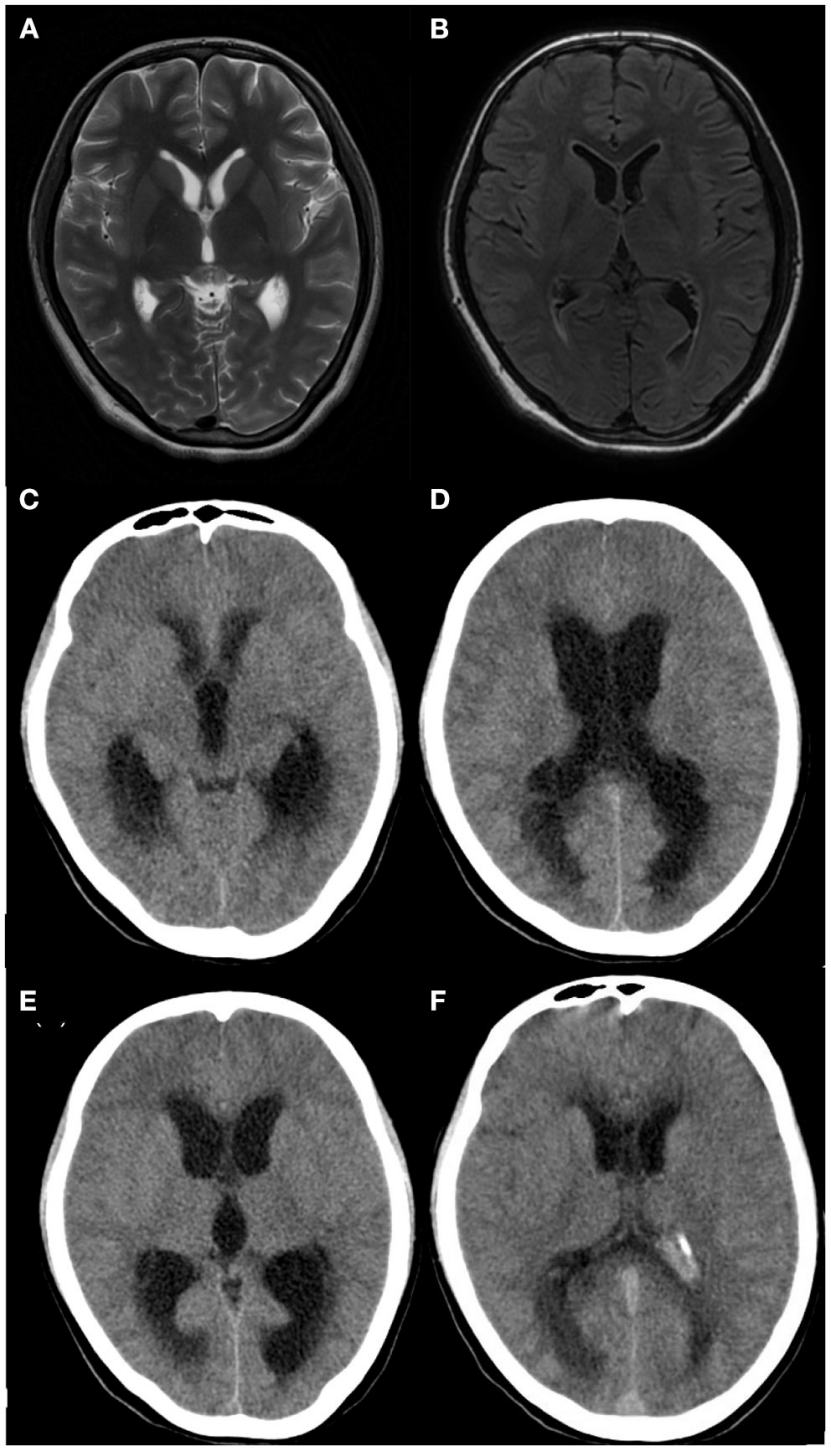
Brain imaging of patient 8. **(A,B)** Brain MRI performed 16 days after clinical onset showing no apparent abnormalities, **(C,D)** Brain CT performed 20 days after clinical onset showing hydrocephalus and parenchymal edema, **(E,F)** Brain CT performed after ventricular-puncture drainage (27 days after clinical onset), Though no significant reduction of hydrocephalus was shown in panel E compared to panel C, decreased hydrocephalus was obvious in panel F compared to panel D.

Computed tomography (CT) or MRI of the brain showed that 50% (4/8) of the children had abnormalities of which 2 cases presented with hydrocephalus and parenchymal edema (Table 2, Figure 1).

### Microbiological Detection and Data of mNGS

Blood and CSF were collected and submitted for culture in all patients who received antimicrobials before hospitalization. Blood cultures were positive in 3 (37.5%) of the 8 patients and the average interval from symptom onset to sampling date was 4.3 days. CSF culture was positive in 5 (62.5%) patients and the mean duration between symptom onset and sample collection was 7.6 days. The mNGS of the CSF was performed in 5 patients. The results of the mNGS were positive in 4 patients and suspected in 1 patient (Table 3).

**Table 3:** Summary of the microbiological investigations in the patients.

**Patient**	**Blood culture**	**CSF culture**	**Specific sequences of mNGS**	**Onset-sample of mNGS(days)**	**Confidence level of mNGS**	**Interval from sample date to diagnosis (days)**
1	Positive	Positive	2	53	Suspected	3
2	Positive	Positive	ND	ND	ND	2
3	Negative	Negative	60	12	Positive/definite	1
4	Positive	Positive	26,446	8	Positive/definite	1
5	Negative	Negative	208	4	Positive/definite	1
6	Negative	Negative	86	10	Positive/definite	1
7	Negative	Positive	ND	ND	ND	1
8	Negative	Positive	ND	ND	ND	1

*CSF, cerebrospinal fluid; NGS, Next-generation sequencing; ND, not done*.

### Anti-infective Therapy and Outcomes

Parenteral antibiotic treatment was administered to all patients (100%) before lumbar puncture (Table 4). After *L. monocytogenes* was confirmed as the pathogenic bacteria and according to the changes of body temperature and CSF indicators (inspection weekly), targeted-antibiotic treatment was adjusted (Table 4). The complications of *L. monocytogenes* infection in our subjects included hyponatremia, hydrocephalus, and hemophagocytic syndrome (Table 4). The subjects were followed up for a minimum of 8 months and a maximum of 3 years. 6 cases had a good prognosis (GOSE = 5) but 2 patients died and neither of these cases underwent autopsy.

**Table 4:** Summary of the anti-infective therapies, complications and outcomes of the patients.

**Patient**	**Empirical antibiotics**	**Targeted antibiotics and course**	**Hospital days**	**Complications**	**Final CSF results**			**Glasgow Outcome Score (GOSE)**
					**WBC count × 10^**6**^/L**	**Protein concentration (g/L)**	**Glucose (mmol/L)**	
1	Meropenem vancomycin	Meropenem (44d) Penicillin (15d) Rifampicin (15d) TMP/SMX (18d) Ampicillin (20d)	60d	N	*17*	150.7	2.51	5
2	Ceftriaxone penicillin	Penicillin and Ceftriaxone (22d) Meropenem and Linezolid (20d)	46d	N	12	210.8	2.45	5
3	Penicillin moxalactam	Penicillin and Moxalactam (26d)	26d	N	12	*542.6*	2.55	5
4	Meropenem ceftriaxone	Meropenem (21d) Linezolid (20d)	21d	N	12	252.3	2.57	5
5	Ceftriaxone	Meropenem (25d) Linezolid (20d)	27d	Hyponatremia	10	310	2.49	5
6	Moxalactam	Meropenem (19d) Vancomycin (9d) Linezolid (14d) Rifampicin (26d) Penicillin (17d)	59d	Hemophagocytic syndrome	10	260.9	2.37	5
7	Moxalactam	Ampicillin (26d) Ceftriaxone (17d) Meropenem (6d)	29d	Hyponatremia hydrocephalus	*20*	*26,753*	*2.09*	1
8	Ceftriaxone	Meropenem and Ampicillin (23d)	25d	Hyponatremia hydrocephalus	7	*745.5*	4.21	1

*TMP/SMX, Trimethoprim-sulfamethoxazole; d, days; N, none; Normal ranges of WBC, protein concentration and glucose of CSF are 0–15 × 10^6^/L, 200–400 g/L, 2.8–4.5 mmol/L*.

## Discussion

In this study, we report LMM in 8 previously healthy children. The clinical manifestations were non-specific and similar to other forms of bacterial meningitis including fever, vomiting, seizures, neck stiffness, headache, and altered mental status (10, 11). Differential diagnosis of meningitis-like syndromes is highly dependent on geographical, seasonal, and age data. Based on blood and CSF analysis, purulent meningitis was diagnosed in cases with relatively high CSF white cell counts (over 15 × 10^6^/L) and low CSF glucose levels (50% blood glucose), (10). In this study, the peripheral blood leukocytes of 3 patients (Patient 2, 7, 8) were close to normal yet the levels of neutrophils and CRP were increased in all cases. Previous reports showed that typical monocyte predominance was different from other general forms of meningitis (12, 13), whereas neutrophilic predominance has been reported in other studies (10, 11, 14–16). These findings may be due to the increases in monocytes due to the presence of *L. monocytogenes* which is a facultative intracellular pathogen. *L. monocytogenes* can enter into non-phagocytic cells or be taken up by phagocytic cells to activate monocyte transformation into macrophages (17–19). Prehospital antibiotic use may induce atypical CSF alterations. Monocyte predominance can make the early diagnosis of LMM in patients challenging and occasionally the disease is misdiagnosed as tuberculous meningitis (20) which is a relatively common disease in northwestern China.

Outbreaks of listeriosis are mostly related to food safety problems (2–4). Sporadic cases are often connected with a history of unclean diets. We considered the diet-related triggers after a definitive diagnosis and found that 4 patients had eaten chilled food several days before onset. However, unfortunately, we did not seek evidence of foodborne *L. monocytogenes* as 3 of the families lived far away and the specimens could not be collected in time. The source of contaminated food in the other 1 patient was also unclear, who attended a boarding school where no similar cases were found during the same period.

Adults or elder children patients frequently complain of gastrointestinal problems (12) yet younger children cannot feedback to their pediatrician. Only patient 8 who was a 16-year-old girl complained of abdominal pain while the other children appeared to have uncharacteristic symptoms such as irritability and weakness. The gut microbiota protects against *L. monocytogenes* infection in immunocompromised hosts (18, 21, 22). Listeria infection should be considered when purulent meningitis patients exhibit a prodrome characterized by gastrointestinal symptoms. In these cases, the chilled food or the gut microbiota had changed in the healthy, immunocompetent children. However, the cases were probably misdiagnosed because of non-specific clinical presentation, physician behaviors, limited diagnostic capacity of the laboratory, and lack of surveillance for listeriosis. Studies have shown that blood or CSF cultures were often negative in these circumstances (10, 11, 23, 24).

Although the positive rate of blood or CSF culture in the group of patients reached 62.5% (5/8), the blood and CSF cultures were negative in the other 3 patients (Patient 3, 5, and 6), diagnosed as LMM according to the positive results of mNGS. Multiple cultures are often needed to detect listeria resulting in a delayed diagnosis (14, 15). mNGS can detect all nucleic acids in a specimen containing the host and microbial sequences by sequencing the total DNA or RNA to identify pathogens. This approach overcomes the limitations of traditional pathogen detection with expanded coverage and improved sensitivity and is less affected by previous antimicrobial exposure and the length of the course of treatment (20). The properties support the use of mNGS delivery in rapid diagnosis. Also, mNGS is helpful in the differential diagnosis of CNS infection.

In this study, patient 1 was diagnosed with LMM by blood and CSF culture (from the grass-roots hospitals) but had recurrent fever after 49 days of specific anti-infection treatment with predominantly monocytes in the CSF. We considered the possibility of tuberculous meningitis and so mNGS of the CSF was performed. *L. monocytogenes* were monitored and not affected by anti-infection treatment. *Mycobacterium tuberculosis* was not detected confirming the diagnosis of LMM. mNGS is helpful for differential diagnosis. The rapid development of sequencing technology has reduced the sample-to-answer turn-around time to <24 h. Also, whole-genome sequencing by NGS facilitates the detection of outbreaks and guides trace-back investigations that can lead to the identification of the sources of infection (2, 4).

The first-line treatment of meningitis frequently includes third-generation cephalosporins and vancomycin to target the most common pathogens (25). However, once LMM has been diagnosed, treatment should be adjusted to ampicillin alone or in combination with aminoglycoside (5, 10, 14). Carbapenems used alone or in combination with aminoglycoside have also shown good results (12, 13). Trimethoprim-sulfamethoxazole (TMP/SMX) was recently described as an effective alternative in CNS listeriosis (13, 16, 20). Aminoglycosides are limited in pediatric cases due to toxicity. All the 8 patients in this study were treated with ampicillin or penicillin combined with other antibiotics such as rifampicin (Patient 1,6), TMP/SMX (Patient 1), Ceftriaxone (Patient 2,7), Moxalactam (Patient 3), Meropenem (Patient 1,2,6,7,8), and Linezolid (Patient 6). Meropenem combined with Linezolid was used to treat 3 patients (Patient 2, 4, 5). The treatment duration of the patients in our study varied from 3 weeks to a maximum of 8 weeks. Currently, there is no general consensus concerning the duration of therapy which varies from 10 days to 8 weeks depending on the severity of the cases (14).

In this study, 3 patients (Patient 5, 7, 8) had hyponatremia and uric acid decline (32–95umol/L, the normal range is 178–381umol/L) early in the disease course, without hypotension and dehydration. Blood sodium increased several days after fluid restriction. Syndrome of inappropriate secretion of antidiuretic hormone (SIADH) was diagnosed based on the above symptoms. After treatment with targeted antibiotics, the neurological symptoms and signs disappeared but the fever had persisted for up to 28 days in Patient 6. The patient had a normal body temperature for 16 days that was accompanied by skin rashes, and reduced peripheral blood leukocyte and neutrophil counts (WBCs 2.86–2.96 × 10^9^/L, neutrophils 0.39–0.68 × 10^9^/L). Also, increased levels of ferritin and hemophilic cells were found on bone marrow biopsy and the patient was diagnosed with the hemophilic syndrome. This is the first reported child with LMM combined with the hemophilic syndrome.

A total of 6 patients (Patient 1–6) were cured without Neurological sequelae, however, 2 patients (Patient 7, 8) died. The clinical symptoms improved after anti-infection treatments and the patients were transferred from the intensive care unit to the general care unit. Patient 7 vomited after being hospitalized for 18 days (body temperature was normal for 14 days). Patient 8 vomited and had a headache after being hospitalized for 14 days (body temperature was normal for 10 days). The patients showed a gradual impairment of consciousness and CT imaging detected hydrocephalus. Listeria meningitis has been described in several case reports as the causative pathogen of hydrocephalus. In these cases, lateral cerebral ventricular puncture might be helpful (15, 16, 26), however, in this study, the lateral cerebral ventricular puncture was implemented and the symptoms were aggravated. The parents abandoned treatment and refused an autopsy. Hydrocephalus may be a factor in the poor prognosis of LMM. Children with LMM should be alerted to the occurrence of hydrocephalus even after clinical improvements.

## Conclusions

*Listeria monocytogenes* is a rare cause of meningoencephalitis in previously healthy and immunocompetent children. However, its clinical presentation is similar to that of other viral or bacterial CNS infections and its course can be rapid and aggressive. Physicians should always consider *L. monocytogenes* as a possible etiologic agent of meningoencephalitis, particularly, in cases that are unresponsive to first-line antibiotic treatment, when gram-positive bacilli are observed in the CSF and when gastrointestinal symptoms occur. mNGS may be useful for prompt diagnosis that is required for antibiotic treatment with ampicillin alone or in combination with carbapenems. Pediatricians need to perform close clinical monitoring of children with *L. monocytogenes*-related hydrocephalus.

## Data Availability Statement

The datasets presented in this study can be found in online repositories. The names of the repository/repositories and accession number(s) can be found below: CNBC [accession: PRJCA007477, https://ngdc.cncb.ac.cn/bioproject/browse/PRJCA007477].

## Ethics Statement

This study was reviewed and approved by the Ethics Committee of the Xi'an Children's Hospital. Informed consent was obtained from the parents or legal guardians of the subjects via signed consent forms.

## Author Contributions

TM and XL contributed to conception and design of the study. TM performed the collation of database, made the tables and figures, and wrote the first draft of the manuscript. HX and XL performed the writing-reviewing and editing. TM, XD, FW, DW, HX, and XL performed the analysis and interpretation of data. XD, FW, and HX modified the language. All authors contributed to manuscript revision, read, and approved the submitted version.

## Funding

This work was supported by the Shaanxi Key Science and Technology Innovation Team Project (CN)[2020SF-006].

## Conflict of Interest

HX was employeed by Hugobiotech Co., Ltd. The remaining authors declare that the research was conducted in the absence of any commercial or financial relationships that could be construed as a potential conflict of interest.

## Publisher's Note

All claims expressed in this article are solely those of the authors and do not necessarily represent those of their affiliated organizations, or those of the publisher, the editors and the reviewers. Any product that may be evaluated in this article, or claim that may be made by its manufacturer, is not guaranteed or endorsed by the publisher.
